# Evidence-based guideline of the European Association of Nuclear Medicine (EANM) on imaging infection in vascular grafts

**DOI:** 10.1007/s00259-022-05769-x

**Published:** 2022-04-04

**Authors:** Chiara Lauri, Alberto Signore, Andor W. J. M. Glaudemans, Giorgio Treglia, Olivier Gheysens, Riemer H. J. A. Slart, Roberto Iezzi, Niek H. J. Prakken, Eike Sebastian Debus, Susanne Honig, Anne Lejay, Nabil Chakfé

**Affiliations:** 1 Department of Medical-Surgical Sciences and of Translational Medicine, Sapienza University of Rome, Nuclear Medicine Unit of S. Andrea Hospital, Via di Grottarossa 1035, 00189 Rome, Italy; 2 Medical Imaging Center, Department of Nuclear Medicine and Molecular Imaging, University of Groningen, University Medical Center Groningen, Groningen, the Netherlands; 3 Clinic of Nuclear Medicine, Imaging Institute of Southern Switzerland, Ente Ospedaliero Cantonale, Bellinzona, Switzerland; 4 Department of Nuclear Medicine and Molecular Imaging, Lausanne University Hospital, Lausanne, Switzerland; 5 Academic Education, Research and Innovation Area, General Directorate, Ente Ospedaliero Cantonale, Bellinzona, Switzerland; 6 Faculty of Biology and Medicine, University of Lausanne, Lausanne, Switzerland; 7 Faculty of Biomedical Sciences, Università Della Svizzera Italiana, Lugano, Switzerland; 8 Department of Nuclear Medicine, Cliniques Universitaires Saint-Luc and Institute of Clinical and Experimental Research (IREC), Université Catholique de Louvain (UCLouvain), Brussels, Belgium; 9 Dipartimento Di Diagnostica Per Immagini, Radioterapia Oncologica Ed Ematologia - Istituto Di Radiologia, Fondazione Policlinico Universitario A. Gemelli IRCCS - Università Cattolica del Sacro Cuore, Rome, Italy; 10 Department of Radiology, University Medical Center Groningen, University of Groningen, Hanzeplein 1, 9713 GZ Groningen, The Netherlands; 11 Department for Vascular Medicine, Vascular Surgery–Angiology–Endovascular Therapies, University Heart & Vascular Center Hamburg, University Hospital Hamburg-Eppendorf, Hamburg, Germany; 12 Department of Vascular Surgery and Kidney Transplantation, University of Strasbourg and GEPROVAS, Strasbourg, France

**Keywords:** Infection diagnosis, Vascular graft infection, Imaging, Recommendations

## Abstract

**Purpose:**

Consensus on optimal imaging procedure for vascular graft/endograft infection (VGEI) is still lacking and the choice of a diagnostic test is often based on the experience of single centres. This document provides evidence-based recommendations aiming at defining which imaging modality may be preferred in different clinical settings and post-surgical time window.

**Methods:**

This working group includes 6 nuclear medicine physicians appointed by the European Association of Nuclear Medicine, 4 vascular surgeons, and 2 radiologists. Vascular surgeons formulated 5 clinical questions that were converted into 10 statements and addressed through a systematic analysis of available literature by using PICOs (Population/problem–Intervention/Indicator–Comparator–Outcome) strategy. Each consensus statement was scored for level of evidence and for recommendation grade, according to the Oxford Centre for Evidence-based Medicine criteria.

**Results:**

Sixty-six articles, published from January 2000 up to December 2021, were analysed and used for evidence-based recommendations.

**Conclusion:**

Computed tomography angiography (CTA) is the first-line imaging modality in suspected VGEI but nuclear medicine modalities are often needed to confirm or exclude the infection. Positron emission tomography/computed tomography (PET/CT) with 2-deoxy-2-[^18^F]fluoro-D-glucose ([^18^F]FDG) has very high negative predictive value but it should be performed preferably at least 4 months after surgery to avoid false positive results. Radiolabelled white blood cell (WBC) scintigraphy, given its high diagnostic accuracy, can be performed at any time after surgery.

**Preamble:**

The European Association of Nuclear Medicine (EANM) is a professional no-profit medical association that facilitates communication worldwide between individuals pursuing clinical and research excellence in nuclear medicine. The EANM was founded in 1985. EANM members are physicians, technologists, and scientists specializing in the research and practice of nuclear medicine. The EANM will periodically define new guidelines for nuclear medicine practice to help advance the science of nuclear medicine and to improve the quality of service to patients throughout the world. Existing practice guidelines will be reviewed for revision or renewal, as appropriate, on their fifth anniversary or sooner, if indicated. Each practice guideline, representing a policy statement by the EANM, has undergone a thorough consensus process in which it has been subjected to extensive review. The EANM recognizes that the safe and effective use of diagnostic nuclear medicine imaging requires specific training, skills, and techniques, as described in each document. Reproduction or modification of the published practice guideline by those entities not providing these services is not authorized. These guidelines are an educational tool designed to assist practitioners in providing appropriate care for patients. They are not inflexible rules or requirements of practice and are not intended, nor should they be used, to establish a legal standard of care. For these reasons and those set forth below, the EANM suggests caution against the use of the current consensus document in litigation in which the clinical decisions of a practitioner are called into question. The ultimate judgement regarding the propriety of any specific procedure or course of action must be made by the physician or medical physicist in the light of all the circumstances presented. Thus, there is no implication that an approach differing from the consensus document, standing alone, is below the standard of care. To the contrary, a conscientious practitioner may responsibly adopt a course of action different from that set forth in the consensus document when, in the reasonable judgement of the practitioner, such course of action is indicated by the condition of the patient, limitations of available resources, or advances in knowledge or technology subsequent to publication of the consensus document. The practice of medicine includes both the art and the science of the prevention, diagnosis, alleviation, and treatment of disease. The variety and complexity of human conditions make it impossible to always reach the most appropriate diagnosis or to predict with certainty a particular response to treatment. Therefore, it should be recognized that adherence to this consensus document will not ensure an accurate diagnosis or a successful outcome. All that should be expected is that the practitioner will follow a reasonable course of action based on current knowledge, available resources, and the needs of the patient, to deliver effective and safe medical care. The sole purpose of this consensus document is to assist practitioners in achieving this objective.

**Supplementary Information:**

The online version contains supplementary material available at 10.1007/s00259-022-05769-x.

## Introduction

The pathogenesis of vascular graft/endograft infection (VGEI) is multifactorial and depends on several aspects: patient’s comorbidities and risk factors, surgical procedures (open vs endovascular approach), and environmental factors. Therefore, the real incidence of infection is difficult to assess [1, 2]. However, it has been estimated that the onset of an infection could complicate the graft insertion in up to 4% of cases, being responsible of high morbidity, mortality rate, and economic burden [1]. In many cases, the explant of an infected graft, revascularization, and long-term antibiotic therapy, represent cardinal aspects for the management of these patients. Nevertheless, reintervention may lead to a mortality of 18–30%. On the other hand, a conservative treatment with prolonged antibiotic regimens may also result in high mortality rate within 2 years if the infection is not completely eradicated [1].

An accurate diagnosis of infection and its extent is crucial for the correct patient management, but a combination of clinical findings, imaging studies, and microbiological tests is usually needed [1].

Since there is no evidence in the literature that vascular grafts used in open reconstructions behave differently from endoprostheses, the European Society for Vascular Surgery (ESVS) considers grafts and endografts as the same entity [2] and, therefore, in this guideline, we used one common definition, regardless of the surgical approach and the graft material (synthetic or biological).

VGEI can be categorized according to the Szilagyi and the Samson classification that specifically considers vascular graft involvement, while the Bunt classification places more emphasis on the extent of graft involvement (Table 1) [3–5]. Furthermore, VGEI can be classified as early (< 4 months) or late (> 4 months) onset, according to the time elapsed from surgery [6].

**Table 1:** Classifications for wound and VGEI with respect to wound infection (Szilagyi, Samson) and to the extent of graft involvement (Bunt) [3–5]

Relationship to post-operative wound infection *(Szilagyi and Samson classifications)*
Szilagyi classification:
• Grade I: cellulitis involving the wound
• Grade II: infection involving subcutaneous tissue
• Grade III: infection involving the vascular prosthesis
Samson classification:
• Group 1: no deeper than dermis
• Group 2: subcutaneous tissue, no direct contact with the graft
• Group 3: body of graft but not anastomosis
• Group 4: Exposed anastomosis, no bleeding, no bacteriemia
• Group 5: Anastomosis involved, bleeding, bacteriemia
Extent of graft involvement *(Bunt classification modified)*
Peripheral graft infection:
• P0 graft infection: Infection of a cavitary graft (e.g. aortic arch; abdominal and thoracic aortic interposition; aortoiliac, aortofemoral, iliofemoral graft infections)
• P1 graft infection: Infection of a graft whose entire anatomic course is noncavitary (e.g. carotid-subclavian, axilloaxillary, axillofemoral, femorofemoral, femorodistal, dialysis access bridge graft infections)
• P2 graft infection: Infection of the extracavitary portion of a graft whose origin is cavitary (e.g. infected groin segment of an aortofemoral or thoracofemoral graft, cervical infection of an aortocarotid graft)
• P3 graft infection: Infection involving a prosthetic patch angioplasty (e.g. carotid and femoral endarterectomies with prosthetic patch closure)
Graft-enteric erosion
Graft-enteric fistula
Aortic stump sepsis after excision of an infected aortic graft

To overcome the numerous shortcomings of current classifications, the MAGIC (Management of Aortic Graft Infection Collaboration) group developed a list of major and minor criteria according to clinical, surgical, radiological, and laboratory findings (Table 2) [1]. Once VGEI is suspected, exhaustive evaluation of the clinical status, comorbidities of the patient, and signs of infection according to MAGIC criteria are recommended [2]. According to this classification, a VGEI is *suspected* in a patient with a single major criterion or at least two minor criteria from different categories. A VGEI is *diagnosed* in the presence of one major criterion plus any other (major or minor) criterion from another category.

**Table 2:** The MAGIC classification [1]

MAJOR CRITERIA		
Clinical/Surgical	Radiology	Laboratory
• Pus (confirmed by microscopy) around graft or in aneurysm sac at surgery • Open wound with exposed graft or communicating sinus • Fistula development, e.g. aorto-enteric or aorto-bronchial • Graft insertion in an infected site, e.g. fistula, mycotic aneurysm, or infected pseudoaneurysm	• Peri-graft fluid on CT scan ≥ 3 months after insertion • Peri-graft gas on CT scan ≥ 7 weeks after insertion • Increase in peri-graft gas volume demonstrated on serial imaging	• Organisms recovered from an explanted graft • Organisms recovered from an intra-operative specimen • Organisms recovered from a percutaneous, radiologically guided aspirate of peri-graft fluid
MINOR CRITERIA		
Clinical/Surgical	Radiology	Laboratory
• Localized clinical features of graft infection, e.g. erythema, warmth, swelling, purulent discharge, pain • Fever ≥ 38 °C with graft infection as most likely cause	Other, e.g. suspicious peri-graft gas/fluid soft tissue inflammation; aneurysm expansion; pseudoaneurysm formation: focal bowel wall thickening; discitis/osteomyelitis; suspicious metabolic activity on [^18^F]FDG PET/CT; radiolabelled leukocyte scintigraphy	• Blood culture(s) positive and no apparent source except graft infection • Abnormally elevated inflammatory markers with graft infection as most likely cause, e.g. erythrocyte sedimentation rate, C-reactive protein, white cell count
VGEI is *suspected* in the presence of one single major criterion OR two/more minor criteria from different categories VGEI is *diagnosed* in the presence of one major criterion AND any criterion (major or minor) from another category		

The main imaging criteria for VGEI diagnosis include the presence of fluids and/or gas around a graft, hypermetabolic activity, or a direct communication between non-sterile sites and a graft.

## Purpose of this document

ESVS has recently published clinical guidelines for the management of patients with VGEI that provide practical recommendations for detecting the infection and illustrate the most appropriate prevention strategies and therapeutic options for each vascular district [2]. Although a section on imaging was also included in ESVS guidelines, they were mainly focused on clinical aspects. Therefore, it emerged the need of a separate document aiming at defining which imaging modality may be preferred at different timepoints of the patient history and in a particular clinical setting. A standardization of the diagnostic strategies, indeed, should be attempted in order to reduce the wide heterogeneity in the approach to the problem and the wide range of reported accuracies of different imaging techniques.

This guideline is designed to assist all practitioners involved in the management of VGEI, by summarizing the state of the art of imaging modalities in the assessment of infective complications after a vascular surgery procedure. We aim at providing evidence-based recommendations, useful for achieving effective and safe medical care.

## Imaging modalities for the assessment of VGEI

Advantages and limitations of the different radiological and nuclear medicine (NM) imaging modalities are summarized in Table 3.

**Table 3:** Summary table on radiological and Nuclear Medicine imaging modalities

Imaging modality	Pros	Cons
CTA	High sensitivity and specificity Easy and cheap to be performed	High radiation exposure Contrast allergy Nephrotoxicity
MRI	Functional and dynamic imaging and tissue characterization No iodinated contrast agents No radiation exposure	High costs Poorly tolerated by the patients Possible risk of nephrotoxicity in patients with impaired renal function after gadolinium injection Better to use 3 T scanners Limited role in clinical practice
^99m^Tc-WBC	High sensitivity and specificity SPECT/CT images improve accuracy Able to discriminate between infection and sterile inflammation also in early phases after surgery Well standardized acquisition protocols and interpretation criteria	Poor availability and medium costs Moderate radiation exposure Often requires late acquisitions (20 h p.i.) Blood manipulation Requires sterile facilities and trained personnel
[^18^F]FDG PET/CT	High sensitivity High-quality images Short length of the exam (2–3 h) Does not need blood manipulation Widely available	Low specificity High false positive rate in early phases after surgery (< 4 months) No standardized interpretation criteria Moderate radiation exposure

### Radiological imaging modalities

Imaging modalities should be able to confirm or exclude peri-graft inflammation, delineate the extent of graft infection (based on fluid or gas extent, presence of anastomotic pseudoaneurysm, partial or total graft involvement, graft-enteric erosion/fistula), plan the correct strategy for revascularization, and to allow accurate imaging-guided biopsy or fluids drainage.

Different radiological modalities are available to diagnose VGEI, being computed tomography (CT)/CT angiography (CTA) the most frequently applied for both intraluminal and extraluminal grafts evaluation. The role of Magnetic Resonance Imaging (MRI), at the moment, is very limited.

#### Computed tomography angiography

CTA is the most commonly used imaging modality, when a VGEI is suspected, due to its wide availability and its short acquisition time. CTA is able to evaluate the vascular anatomy and related pathology, the graft involvement as well as the involvement of adjacent structures and infection-related complications [7]. This requires a high spatial resolution CT acquisition protocol, using a 64 multi detectors or Dual Source CT scanner (DSCT), with submillimetre detectors, and thin reconstruction sections. CT images are usually acquired before and after intravenous iodinated contrast medium (CM) administration (high concentration CM preferred; CM volume of approximately 50-80 mL, using a 3-4 mL/s injection flow rate), allowing the evaluation of both arterial and delayed-phases findings as well as the anatomic relationship of the native vessels, the graft, and peri-graft structures [7]. Arterial phase images can be obtained with bolus tracking, using a threshold of 150HU and a diagnostic delay of 5 s; a 60-s delayed phase is usually preferred. For DSCT scanners, in case of dual-energy acquisitions, on the basis of absolute CT attenuation values and relative modifications at low and high kVp, iodine can be removed from the data to create a virtual unenhanced image.

Serial CTA scans may show changes in the peri-graft tissues, thus being able to differentiate a VGEI from post-surgical inflammation [8]. Aneurysm expansion may lead to erosion into bowel, fistulization, and infection. Contrast extravasation from the aorta into the bowel is a rare finding but provides a definitive diagnosis of VGEI. Adherence of the thickened bowel wall to the sac wall and the stranding are clues to the diagnosis, strengthened by the presence of gas in the aneurysm sac.

#### Magnetic resonance imaging

MRI plays a very modest role in the diagnostic investigation of VGEI in research field and has no place in clinical routine. Particularly, during the last 20 years, no significant improvements in MRI techniques, specifically for VGEI detection, have been made due to the wider availability of superior imaging techniques, such as CTA, which is able to depict cardiovascular structures with higher temporal and spatial resolution [9, 10]. Potential advantages of MRI include functional and dynamic imaging and tissue characterization (oedema, fluid, fibrosis). Additionally, no iodinated contrast is needed for MRI, and there is no radiation exposure. In recent years, there is growing interest in tissue characterization using T1 and T2 mapping to quantify myocardial fibrosis, protein, and oedema content in various diseases, including infection and inflammation [11, 12]. Nevertheless, these quantitative imaging techniques are not validated for diagnosing VGEI yet. Either T1 and T2 weighed imaging [13], or the slightly superior short tau inversion recovery (STIR) technique, are the main sequences commonly used [14]. Other limitations of MRI include the need of high field strength to acquire a diagnostic image resolution with adequate signal and contrast to noise ratio for detection of subtle peri-graft fluid collections. MRI has longer examination length, limited availability, and other common contra-indications like claustrophobia. Nowadays, almost all endovascular grafts are MRI compatible, however, they may cause artefacts, thus limiting image analysis, especially the stainless steel grafts. Moreover, the use of gadolinium contrast to depict vasculature is associated with a negligible, but existing, risk of nephrotoxicity in patients with impaired renal function, combined with unelucidated effects of gadolinium deposition in the brain [15].

At present, Magnetic Resonance Angiography (MRA) can visualize the aortic anatomy with high accuracy in a modern electrocardiography (ECG) gated free-breathing phase contrast 3D volume, which can be reconstructed to any viewing plane with its isotropic voxel resolution. This technique is superior to the inherently untriggered contrast bolus MRA with its movement artefacts close to the aortic root [15]. However, the aortic wall thickness imaged with this modern technique is 1–2 voxels thick and, therefore, subtle wall thickening and tissue characterization, such as oedema or protein load, are not easily detectable. For this assessment, cross-sectional imaging is needed but it is a more laborious approach where aortic flow information is still more reliable and valuable in this context than the information acquired by assessing the vascular wall [16].

### Nuclear medicine imaging modalities

The discrimination between infection and sterile inflammation is not always easily achievable with radiology, however, it is crucial to correctly diagnose VGEI [17, 18]. NM modalities have shown high diagnostic accuracy for VGEI, thus providing complementary tools to morphological imaging.

#### White blood cell scintigraphy

Radiolabelled autologous white blood cell (WBC) scintigraphy, with ^111^Indium [^111^In]-oxine or 99-metastable Technetium [^99m^Tc]-exametazime (HMPAO), offers high specificity for the detection of infection [19, 20]. Considering the kinetics of WBC accumulation in infected abdominal vascular grafts, two time-point image acquisition—early, at 30 min, and delayed, at 2-3 h post-injection (p.i.) using time corrected for decay protocols, can be sufficient for imaging intra-abdominal VGEI, thus avoiding interference of non-specific bowel activity that starts accumulating after 3–6 h p.i. A late image time-point acquisition (20 h p.i.), however, can be useful to better visualize slow kinetic infective processes, such as abscesses and fistulae, or in case the 3-h scan is not conclusive.

In some cases, it has been suggested to start the imaging protocol with a 5 min dynamic acquisition after bolus injection, to image the vascular tree, aneurysms, or obstructions [21, 22].

Hybrid imaging, with single-photon emission computed tomography co-registered with CT (SPECT/CT) acquisitions, is an integral part of conventional WBC scintigraphy and it is, therefore, mandatory. It allows a better localization of suspected foci of uptake and an accurate evaluation of its extent, thus reducing equivocal cases and increasing diagnostic accuracy of planar images [21–24].

For SPECT/CT images, times corrected for decay protocols are not compulsory, unless quantitative comparison is required [21]. A SPECT/CT at 2–3 h p.i. (usually 15–20 s/step, one step/6°) avoids non-specific gastro-intestinal activity and requires shorter acquisition time, but a SPECT/CT at 20 h p.i. (usually 30–40 s/step, one step/6°) may have the advantage of confirming the pathological uptake observed at 3 h, thus depicting the exact location of fistulae and abscesses.

For peripheral grafts, standard acquisition protocols with three time points (early, at 30 min, delayed, at 3–4 h, and late, at 20–24 h) can be adopted.

**Fig. 1:**
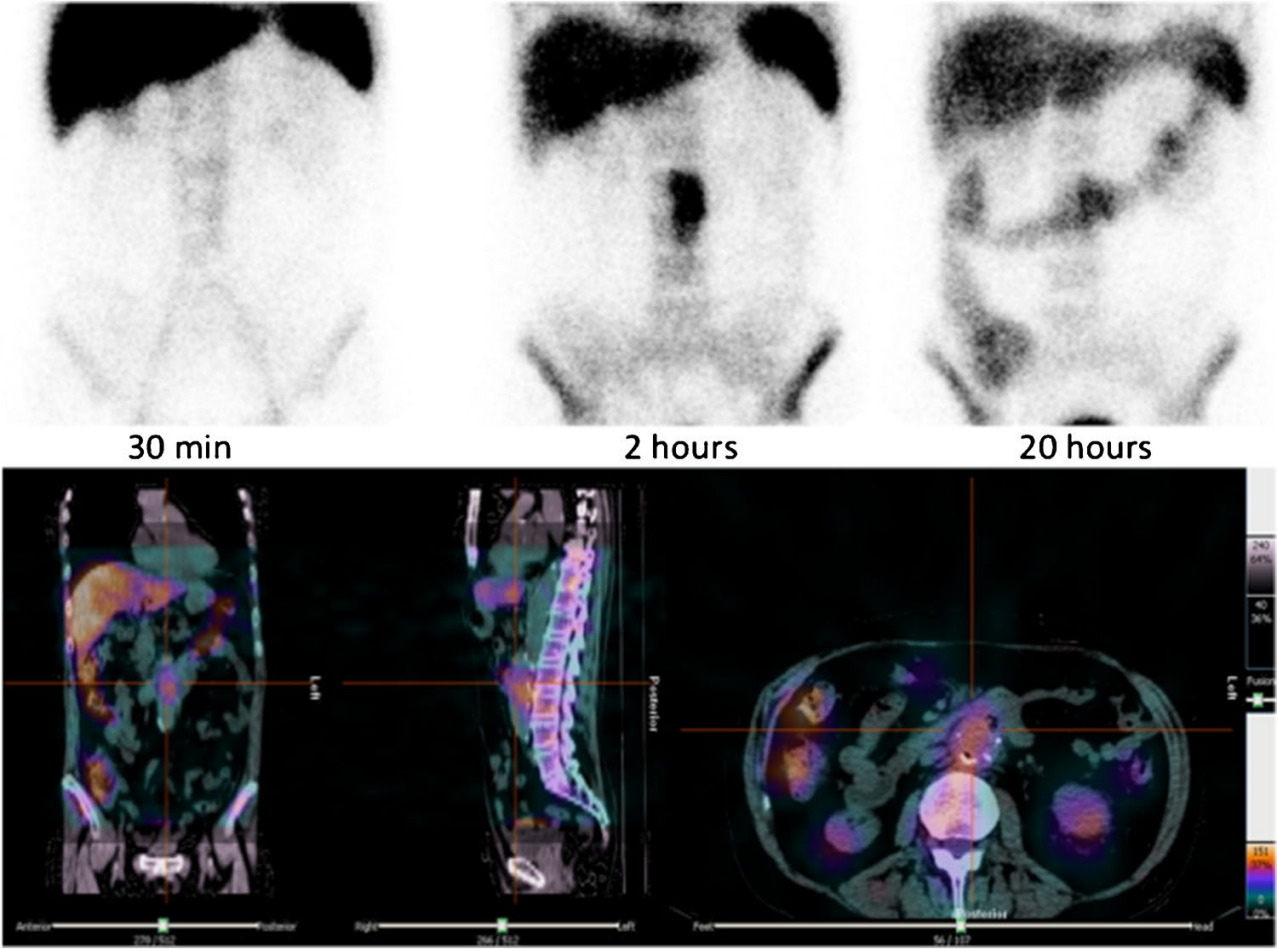
Planar images of WBC scintigraphy, acquired at 30 min, 2 and 20 h p.i., show an increased uptake over time, that is consistent with an infection of abdominal graft (upper panel). SPECT/CT images, acquired 2 h p.i., allow to accurately localize the uptake and to evaluate its extent (lower panel)

Once acquired, the images have to be correctly displayed with a total count intensity scale using the same threshold in order to ensure a correct interpretation of the examination and to limit the observer bias [25, 26]. An increased accumulation of radiolabelled WBC over time, in terms of extent and/or intensity, is consistent with an infection (Fig. 1); conversely, a sterile inflammation shows a decreased or stable uptake over time.

### *[*^*18*^*F]FDG PET/CT*

2-deoxy-2-[^18^F]fluoro-D-glucose ([^18^F]FDG) positron emission tomography/CT (PET/CT) has several advantages over planar images of labelled WBCs considering its wide availability and the shorter length of the exam without requiring potentially infected blood manipulation. However, this modality usually provides higher rate of false positive findings, especially in the post-surgical period, since it cannot always distinguish the physiological sterile inflammation from an infection or thrombosed grafts. Increased [^18^F]FDG uptake in vascular grafts can persist for a long time, even years, after surgery depending on the material used [27–30].

Several interpretation criteria have been proposed, although not universally accepted. Some authors suggest a four- or five-point visual scale to diagnose VGEI [31, 32], others consider the intensity and focality of uptake as criterion of a positive scan [28, 33]. The contribution of a semi-quantitative assessment with standardized uptake values (SUV) or target-to-background (T/B) ratios has also been explored, but its role still remains controversial [30, 34]. Indeed, no definite threshold, able to differentiate an infection from a sterile inflammation, has been identified.

## Methodology

### Working group and strategy

From several joint symposia and from the available literature, it emerged the need of an evidence-based guideline for imaging VGEI. To this aim, the EANM of Inflammation & Infection Committee created a working group together with radiologists and vascular surgeons. Vascular surgeons formulated some clinical questions that should be addressed by imaging. These clinical questions were the starting point for the definition of several statements that were used to perform a literature search based on the PICO (Population/problem–Intervention/Indicator–Comparator–Outcome) strategy. Papers of interest were graded by level of evidence and used to provide final recommendations, which were graded according to the Oxford Centre for Evidence-based medicine criteria (OCEBM) [35, 36]. Selected papers for each statement were analysed and scored by all participants and, after several revisions, all the members of the writing group approved the final version of this document.

### Clinical questions

Clinical presentation of VGEI can be highly suggestive for infection, particularly in early post-operative infection (< 4 months). However, late infections can be misdiagnosed, especially for abdominal or thoracic VGEI, due to low-grade clinical and/or laboratory criteria.

The recently published ESVS guidelines, including the contribution of NM physicians and radiologists, proposed an algorithm for imaging VGEI, depending on the location of the graft [2]. CTA is the first-line imaging modality in suspected VGEI. This is due to its wide availability, fast execution, and the possibility to identify life-threatening complications. Functional imaging with [^18^F]FDG PET/CT and/or WBC scintigraphy with SPECT/CT can complete the work-up in case of negative or doubtful results and persisting clinical suspicion.

Even if vascular surgeons are relatively familiar with CTA findings (peri-graft fluid, ectopic gas, and soft tissue enhancement), the interpretation of NM images is more difficult.

The most important questions for the vascular surgeon when a VGEI is suspected are:

- How to differentiate a physiological from a pathological [^18^F]FDG uptake, especially in the early post-operative period?
- Is it possible to assess if the whole graft or only a part of the graft is infected?
- Is it possible to differentiate severe from low-grade infections?
- Is it possible to differentiate between microbial infection and non-microbial peri-graft reaction?
- Is it possible to differentiate between an infection of the graft and an infection of the native aortic wall?

### Statements

Based on the above questions, ten statements were defined, aiming at providing evidence-based answers. Each consensus statement is followed by comments derived from analysis of the literature and by a conclusive recommendation that could be relevant, in daily practice, for patient’s management.

### Literature search

An extensive literature search for each statement was carried out in PubMed/Medline and Scopus databases, from January 2000 to December 2021. Cross-search with included references in the retrieved articles and hand search were also performed seeking for further evidence. Search terms were defined in agreement with all members of the writing group. Inclusion of papers in each statement was based on a PICO question that was converted into a search strategy, as described by OCEBM [35, 36].

Case reports, abstracts, papers with less than 10 patients and not published in English language were excluded. Systematic reviews were included.

Search results for each statement are summarized in Appendix (supplementary material).

### Scoring system and recommendation grading criteria

All included papers per statement were thoroughly read and analysed. A “level of evidence”, for each paper, was provided in consensus with all delegates according to the documents provided by OCEBM [35, 36] (Table 4a). Each consensus statement is followed by a level of evidence, defined by the average of the levels of evidences of each included paper, and a short comment derived from analysis of the relevant literature. At the end of each statement, a final recommendation is also provided and graded, again in agreement with all delegates, based on the average of paper scores (Table 4b).

**Table 4:** Level of evidence for references (a) and grades of recommendation (b) according to OCEBM [35, 36]

a					
Question	Level 1 (a, b, c)	Level 2 (a, b)	Level 3 (a, b)	Level 4	Level 5
Diagnosis	Systematic reviews with homogeneity^1^ of Level 1 studies; CDR^2^ with studies from different clinical centres; validating cohort studies^3^ with good^4^ reference standards; CDR^2^ in one clinical centre; absolute SpPins and SnNouts^5^	Systematic reviews with homogeneity^1^ of Level 2 studies; Exploratory^3^ cohort studies with good^4^ reference standards; CDR^2^ after derivation, or validated only on split-sample^6^ or databases	Systematic reviews with homogeneity^1^ of Level 3 studies; Non-consecutive studies or without consistently applied reference standards	Case–control studies, or “poor or non-independent” reference standard	Expert opinion without explicit critical appraisal, or based on physiology, bench research, or “first principles”
b					
Grade of recommendation	Definition				
A	Consistent level 1 studies				
B	Consistent level 2 or 3 studies or extrapolations from level 1 studies				
C	Level 4 studies or extrapolations from level 2 or 3 studies				
D	Level 5 evidence or troublingly inconsistent or inconclusive studies of any level				

^1^By homogeneity, we mean a systematic review that is free of worrisome variations (heterogeneity) in the directions and degrees of results between individual studies. Not all systematic reviews with statistically significant heterogeneity need to be worrisome, and not all worrisome heterogeneity need to be statistically significant. Studies displaying worrisome heterogeneity should be tagged with a “-” at the end of their designated level.

^2^Clinical Decision Role (these are algorithms or scoring systems that lead to a prognostic estimation or to a diagnostic category).

^3^Validating studies test the quality of a specific diagnostic test, based on prior evidence. An exploratory study collects information and trawls the data (e.g. using a regression analysis) to find which factors are significant.

^4^Good reference standards are independent of the test and applied blindly or objectively to applied to all patients. Poor reference standards are haphazardly applied, but still independent of the text. Use of a non-independent reference standard (where the “test” is included in the “reference”, or where the “testing” affects the “reference”) implies a Level 4 study.

^5^An “Absolute SpPin” is a diagnostic finding whose Specificity is so high that a positive result rules-in the diagnosis. An “Absolute SnNout” is a diagnostic finding whose sensitivity is so high that a negative result rules-out the diagnosis.

^6^Split-sample validation is achieved by collecting all the information in a single tranche, then artificially dividing this into “derivation” and “validation” samples.

### Update procedure

It is an aim of the EANM to revise this document when important and new evidences will be available in the literature, or at least every 5 years.

## Consensus statements

1. ***CTA represents a valuable tool in diagnosing VGEI, despite a wide range of sensitivity and specificity.***

**Level of evidence: 2**

CTA is widely considered as the first imaging modality in case of suspected VGEI, being able to detect peri-graft air or fluid collections as well as abscesses into adjacent soft tissues. Moreover, it provides useful information on the assessment of complications such as sepsis, disruption of infected anastomotic portions, development of pseudoaneurysm or a *“*de novo*”* type 3 endoleak, peripheral embolization of infected thrombi, reinfection of the grafts, and life-threatening complications (e.g. aorto-oesophageal/enteric, aorto-bronchial, and arterio-ureteral fistula).

However, its diagnostic performance mainly relies on the grade of VGEI, showing a high rate of false negatives in low-grade infective processes [22].

In a systematic review and meta-analysis performed in patients with suspected VGEI, the pooled sensitivity and specificity of CTA were 67% and 63%, respectively [7]. Husmann et al. reported a high diagnostic accuracy for CTA (78.3 and 86.9%, respectively, for the two involved readers). The sensitivity ranged from 92.3 to 100% and the specificity from 50 to 80% [37]. These results are in line with those provided by Fukuchi (79%, 64%, and 68% for accuracy, sensitivity, and specificity, respectively) and Saleem (values ranging between 7 and 100% for specificity and 10–100% for sensitivity, according to the different CT parameters analysed) [31, 38].

CTA can also be useful, in association to Doppler-ultrasound, in the diagnostic work-up of extracavitary VGEI, as reported by Sapienza et al. in case of infection of prosthetic patch after femoral angioplasty presenting with a pseudoaneurysm of the femoral region, even though there is no information on its accuracy [39].

In conclusion, CTA represents a valuable tool in diagnosing VGEI, even though a wide range of sensitivity and specificity exist, mainly related to the grade of graft infection [22]. Indeed, CTA has high accuracy (90–100%) in high-grade VGEI but it shows only moderate accuracy (70–90%) in low-grade processes and post-operative setting. In these situations, a high rate of false negative scans is reported, since some radiological signs can be common to both infection and inflammation—the latter being common in the post-operative setting [1]. Therefore, in case of negative or equivocal CTA findings and persisting strong suspicion of VGEI, the use of NM modalities is recommended.

Nevertheless, due to the relatively low accuracy of CTA in differentiating sterile post-surgical inflammation from infection, a positive CTA should be always confirmed by a NM examination, especially when clinical presentation or laboratory findings are vague (presence of only minor MAGIC criteria) and the probability of infection is low.

**Table:** 

Grade B	Recommendation 1
	Due to low sensitivity and moderate accuracy of CTA for low-grade infective processes, NM modalities are recommended in negative or equivocal CTA and persisting suspicion of VGEI

2. ***MRI has low accuracy for VGEI.***

**Level of evidence: 4**

MRI plays a very modest role in the diagnostic investigation of VGEI in research and has no place in clinical routine. MRI has low accuracy for diagnosing VGEI, especially for chronic or late infections (> 3–6 months) with subtle fluid collections and receded oedema [9, 10]. Only a few small sample comparative studies demonstrated that the presence of fluid collection after 6 post-operative weeks on T1 and T2 imaging could suggest VGEI better than CT [17]. A systematic review of diagnostic MRA performance showed good positive (95%) and negative (80%) predictive value for endoleaks, however, the diagnostic performance of MRA for VGEI was not reported [40]. In 2016 the American Heart Association published a scientific statement on VGEI, mycotic aneurysms, and endovascular infections, reporting a 85–100% sensitivity and a 97–100% specificity [41]. Consequently, MRI is recommended if CT is nondiagnostic to differentiate from hematoma, inflammation, or infection. These authors further state that MRI/MRA can detect mycotic aneurysm, bleeding, and enteric fistula thanks to the good soft tissue resolution, based on the literature published in 1997 and before.

Modern ECG gated free-breathing phase contrast 3D volume MRA technique has improved significantly in recent years and is expected to be superior to MRA. Indeed, this 3D volume technique with its proton-density like weighing, is able to depict tissue boundaries with much higher definition and precision, since it lacks the motion artefacts contrast bolus observed with MRA. Nevertheless, to our best knowledge, there is no literature regarding the diagnostic performance of this technique in VGEI.

In recent years, only few studies on MRI and MRA have been published and provided varying results in different stages of VGEI. ECG gated free-breathing phase contrast 3D volume MRA could be a good alternative and awaits its evaluation in a clinical research setting.

**Table:** 

Grade C	Recommendation 2
	MRI has low accuracy for diagnosing VGEI, especially for chronic or late infections, and is not recommended as first imaging choice

3. ***WBC scintigraphy has high diagnostic accuracy in differentiating VGEI from post-surgical inflammation.***

**Level of evidence: 2**

Post-surgical inflammation, especially in early post-operative phase, represents the most important diagnostic challenge. It could complicate the interpretation of different imaging modalities, thus limiting their ability to properly diagnose an infective process. This aspect is particularly important for the assessment of VGEI, in which post-surgical inflammation may persist for many years after surgery, thus representing a confounding factor that could lead to a wrong management of the patient. Low-grade infections represent another insidious aspect since clinical manifestations may be ambiguous and both biochemical parameters and conventional imaging modalities may fall.

The ability of radiolabelled WBC scintigraphy in differentiating an infective process, even of low-grade, from a sterile inflammation is remarkable. However, its accuracy strongly relies on the application of well-standardized acquisition protocols and interpretation criteria that have been published by EANM in the last decade [19–21].

For this PICO, 80 papers were retrieved and 8 studies were finally included [7, 22, 42–45].

In 2006, Liberatore et al. investigated the accuracy of radiolabelled WBC scintigraphy in diagnosis of VGEI within 1 month from surgery reporting no false positive results in the early post-operative phase after endovascular approach. Therefore, they concluded that this imaging modality is able to accurately differentiate between infection and sterile inflammation [42]. Similarly, in the study of de la Rubia-Marcos et al., radiolabelled WBC scan with SPECT/CT, showed significantly higher sensitivity, specificity, positive and negative predictive value than CT (respectively, 100%, 95%, 91%, and 100% vs 62.5%, 76%, 55.6, and 81.3%), being able to accurately rule out an infection in early post-operative period and to correctly diagnose a VGEI in later phases. In this study, CT showed high number of false negative scans both in early infections, where the findings were wrongly interpreted as post-surgical changes, and in late infections, where CT did not show any alteration [43]. In another recent comparative study by Puges et al., radiolabelled WBC scan showed higher accuracy than [^18^F]FDG PET/CT, even in patients under antibiotic treatment and in thrombosed graft infection [44].

This also clearly emerges from a recently published systematic review and meta-analysis comparing CTA, radiolabelled WBC scintigraphy with and without SPECT/CT, [^18^F]FDG PET, and PET/CT. The reported pooled sensitivity, specificity, positive pre-test probability, and negative post-test probability of radiolabelled WBC reached the highest values, especially if performed with SPECT/CT acquisitions [7]. Indeed, several authors explored the role of SPECT/CT confirming its added value in the assessment of VGEI. In particular, the studies published by Khaja and Erba reported very high accuracies, being, respectively, 80% for ^111^In-WBCs [45] and 100% for ^99m^Tc-WBCs in detecting low-grade infections [22].

Conversely, Shahidi and Muhammad did not reach similar conclusions [46, 47]. However, the acquisition protocols and interpretation criteria adopted in these studies did not adhere to EANM guidelines, thus justifying the low diagnostic performance.

Consequently, despite some differences in the reported accuracy of this imaging modality still exist in the literature, being lower in the studies that did not follow EANM guidelines, there is a strong evidence to conclude that radiolabelled WBC scintigraphy + SPECT/CT allow the differentiation between post-surgical sterile inflammation and infective processes.

**Table:** 

Grade B	Recommendation 3
	WBC scan with SPECT/CT may be used to accurately differentiate an infection from a sterile inflammation

4. ***Antibiotic therapy has no influence on diagnostic accuracy of WBC scintigraphy in detecting VGEI.***

**Level of evidence: 4**

The issue of whether antimicrobial treatment may have an influence on diagnostic accuracy of WBC scan is still matter of debate.

For this PICO, 73 papers were retrieved but only 3 studies were finally selected [22, 43, 44]. However, no studies directly comparing patients with and without antibiotic treatment for VGEI exist. Moreover, the relative low numbers of patients included in each study, the lack of information about treatments, class of antibiotic and their duration, does not allow to draw definite conclusions on this issue and therefore, information could be only extrapolated from available data.

In the studies published by Erba et al. and by de la Rubia-Marcos et al. the authors did not observe any false negative scan at WBC scintigraphy, even in patients that were receiving antibiotic treatment [22, 43], thus concluding that the accuracy would not be affected by ongoing antimicrobial therapy. This aspect has been more deeply investigated in a recent retrospective study comparing WBC scintigraphy and [^18^F]FDG PET/CT in 39 patients with suspected VGEI. Antibiotic treatment was started before NM imaging in 16 (41%) patients. Overall, WBC scan showed significantly higher diagnostic value compared to [^18^F]FDG PET/CT. In patients that were receiving antibiotics, the sensitivity of both imaging modalities was not altered, but WBC scintigraphy showed a significantly higher accuracy than [^18^F]FDG PET/CT [44].

However, since chemotaxis and cytokines-induced recruitments of leukocytes into the infection site are two crucial aspects for the accuracy of WBC scintigraphy, it is conceivable that some types of antibiotics could impair the ability of radiolabelled WBCs to migrate into infected site. For this reason, there is a general feeling among the NM community that WBC scintigraphy should be preferably performed at least 2 weeks after therapy discontinuation, or, even better, before starting the treatment.

In summary, there is not sufficient evidence in the literature to reach a definitive conclusion on the impact of antibiotic treatment on the accuracy of WBC scintigraphy in VGEI. The choice to stop or continue the therapy should be, therefore, clinically evaluated in the single case and jointly discussed with the referring physicians.

It would be interesting to address, in future, direct and indirect effects of different antimicrobial agents on different aspects of immune response to infections, and on WBCs in particular.

**Table:** 

Grade C	Recommendation 4
	No definitive conclusion can be reached in the literature to withdraw or continue antibiotic therapy prior to WBC scintigraphy. The single clinical case should be discussed multidisciplinary

5. ***[***^***18***^***F]FDG PET/CT has high sensitivity for diagnosing VGEI.***

**Level of evidence: 2**

Several original studies (prospective and retrospective observational cohort studies but no single randomized controlled trial), systematic reviews, and meta-analyses have addressed the sensitivity of [^18^F]FDG PET/CT in patients with suspected VGEI [7, 48–50]. The main findings of the available systematic reviews and meta-analyses are summarized in supplementary material (Supplementary Table 1).

Overall, the pooled sensitivity of [^18^F]FDG PET/CT to detect VGEI was high, ranging from 89 to 98%. Conversely, pooled specificity was very variable, ranging from 59 to 81% [7, 48–50].

It is worthwhile to mention that the original studies were quite heterogeneous and lacking in standardization. Factors contributing to this heterogeneity were among others: characteristics of included patients, grade of infection, characteristics of vascular grafts (location, material used, surgical technique), time intervals between surgery and imaging, use of antibiotics prior to PET/CT, differences in PET/CT cameras, acquisition protocol, image analysis and interpretation, reference standard used and study quality. All these factors may theoretically affect the sensitivity of [^18^F]FDG PET/CT in detecting VGEI [7, 48–50].

Reinders Folmer et al. demonstrated that hybrid [^18^F]FDG PET/CT increases the diagnostic accuracy by reducing the number of false positive and false negative results compared to stand alone PET [7]. It is, indeed, well known that the evaluation of co-registered CT scan is an integral part for the interpretation of a PET/CT study since it is able to provide information about the structure involved in the pathological process (graft, aneurysmatic sac, peri-graft tissues). However, diagnostic accuracy also relies on the interpretation criteria adopted in different studies [48, 50–52]. Two meta-analyses demonstrated that all three different [^18^F]FDG PET/CT interpretation criteria, (i) visual [^18^F]FDG intensity uptake, (ii) visual [^18^F]FDG uptake pattern, and (iii) semi-quantitative analysis using SUVmax, yielded a high sensitivity.

Nevertheless, visual [^18^F]FDG uptake intensity is less specific compared to pattern analysis or SUVmax (59% vs 81% and 77%, respectively) [48, 50]. Overall, data available in the literature demonstrate that heterogeneous, (multi)focal, and high [^18^F]FDG uptake around the vascular graft is consistent with VGEI. Therefore, the diagnostic accuracy may be enhanced by combining the assessment of [^18^F]FDG uptake pattern and intensity [48, 50].

In addition to a pure visual assessment, semi-quantitative parameters can be used to detect VGEI including SUVmax values, corresponding to the hottest [^18^F]FDG signal, or T/B ratios, by dividing the SUVmax of the graft uptake by the SUVmean of a reference region (e.g. liver or blood pool). To date, most of the published studies used the SUVmax as preferred semi-quantitative parameter with only few data available for T/B ratios. The reported sensitivities and specificities of SUVmax analysis ranged between 95 and 98% and from 77 and 80%, respectively [48, 50]. However, a standardized SUVmax cut-off value able to distinguish, with high accuracy, VGEI from non-infected grafts has not been defined yet. Moreover, SUVmax values depend on several characteristics including the type of tomographs, incubation time, reconstruction algorithms, etc., thus making this parameter not reproducible in different centres [50].

In conclusion, solid data clearly demonstrate the high sensitivity of [^18^F]FDG PET/CT in diagnosing VGEI, regardless of the interpretation criteria (visual or semi- quantitative) used. Therefore, it could be performed to rule out the infection. A higher degree of accuracy may be achieved by combining several interpretation methods [48, 50]. Finally, standardization of [^18^F]FDG PET/CT acquisition and interpretation is warranted to reduce study heterogeneity and to allow a comparison between studies.

**Table:** 

Grade B	Recommendation 5
	[^18^F]FDG PET/CT has high sensitivity in diagnosing VGEI, regardless of the interpretation criteria used. Therefore, it can be used to rule out the infection

6. ***Antibiotic therapy may influence the diagnostic accuracy of [***^***18***^***F]FDG PET/CT in detecting VGEI.***

**Level of evidence: 3**

[^18^F]FDG PET/CT evaluation can be influenced by several factors such as diabetes mellitus and the use of antibiotics, as reported in recent systematic reviews and meta-analyses [48, 50]. However, a complete overview on this topic cannot be provided due to the lack of specific information on antibiotic treatments in many papers [7, 49, 50]. Indeed, the use of antimicrobials, the class, and the duration of the treatment are scarcely mentioned and not always linked to the results of [^18^F]FDG PET/CT or to the gold standard for the diagnosis, therefore, the definition of false positives and false negatives is problematic and arbitrary [48, 50]. In a prospective cohort study, Sah et al. demonstrated that the diagnostic performance of [^18^F]FDG PET/CT was higher in patients without previous antimicrobial treatment compared to patients with ongoing therapy [53]. Overall, diagnostic accuracy of [^18^F]FDG PET/CT could be increased by performing the scan prior to antimicrobial treatment, nevertheless clinical presentation may be acute in some patients with VGEI, especially in the presence of highly virulent pathogens, requiring immediate antimicrobial treatment [48, 50]. Once an antibiotic treatment has started, a declining metabolic activity in infections is expected. This might affect the sensitivity of [^18^F]FDG PET/CT [48]. Long-term antibiotic treatment could increase the number of false negative findings at both [^18^F]FDG PET/CT and microbiology, thus resulting in inadequate treatment of infected patients [7, 50].

However, the influence of antibiotics on the number of false negative results at [^18^F]FDG PET/CT remains under investigation [46].

Interestingly, recent studies suggested a potential role of [^18^F]FDG PET/CT in monitoring treatment response in patients with VGEI [53–56]. In particular, a prospective cohort study by Husmann et al. demonstrated decreasing SUVmax values over time in patients undergoing antibiotic therapy and that the capability to detect residual infection by [^18^F]FDG PET/CT was not hampered by antimicrobial therapy [56].

In conclusion, even though literature data concordantly underline the possible influence of antibiotic therapy on the diagnostic accuracy of [^18^F]FDG PET/CT in VGEI, more high-quality studies are needed to further investigate this topic. In daily practice, the decision to stop or not the antibiotic treatment should depend on the clinical conditions of the patient and should derive from a multidisciplinary discussion with the referring physicians.

**Table:** 

Grade C	Recommendation 6
	More robust studies are needed to confirm this effect. The choice to stop or continue antibiotic treatment depends on the single clinical case, preferably discussed within a multidisciplinary team

7. ***Focal [***^***18***^***F]FDG uptake is a reliable diagnostic tool to diagnose an infection.***

**Level of evidence: 2**

In the last decades [^18^F]FDG PET/CT has emerged in the work-up of VGEI, as demonstrated by the large amount of available literature on this topic. Nevertheless, a remarkable variability in the interpretation criteria and parameters (focal pattern, visual scoring scales, SUVmax, T/B ratios, dual-time-point evaluation) adopted among the studies, is evident. Indeed, a major concern in the diagnosis of VGEI with [^18^F]FDG PET/CT is image interpretation and several criteria have been proposed for differentiating the infection from sterile post-surgical inflammation or foreign body reaction.

For this PICO, 109 papers were retrieved and 26 studies were finally selected [28–33, 37, 38, 44, 49, 52–67]. Interestingly, 17 of them were also included in four recent meta-analyses [7, 48–50].

**Fig. 2:**
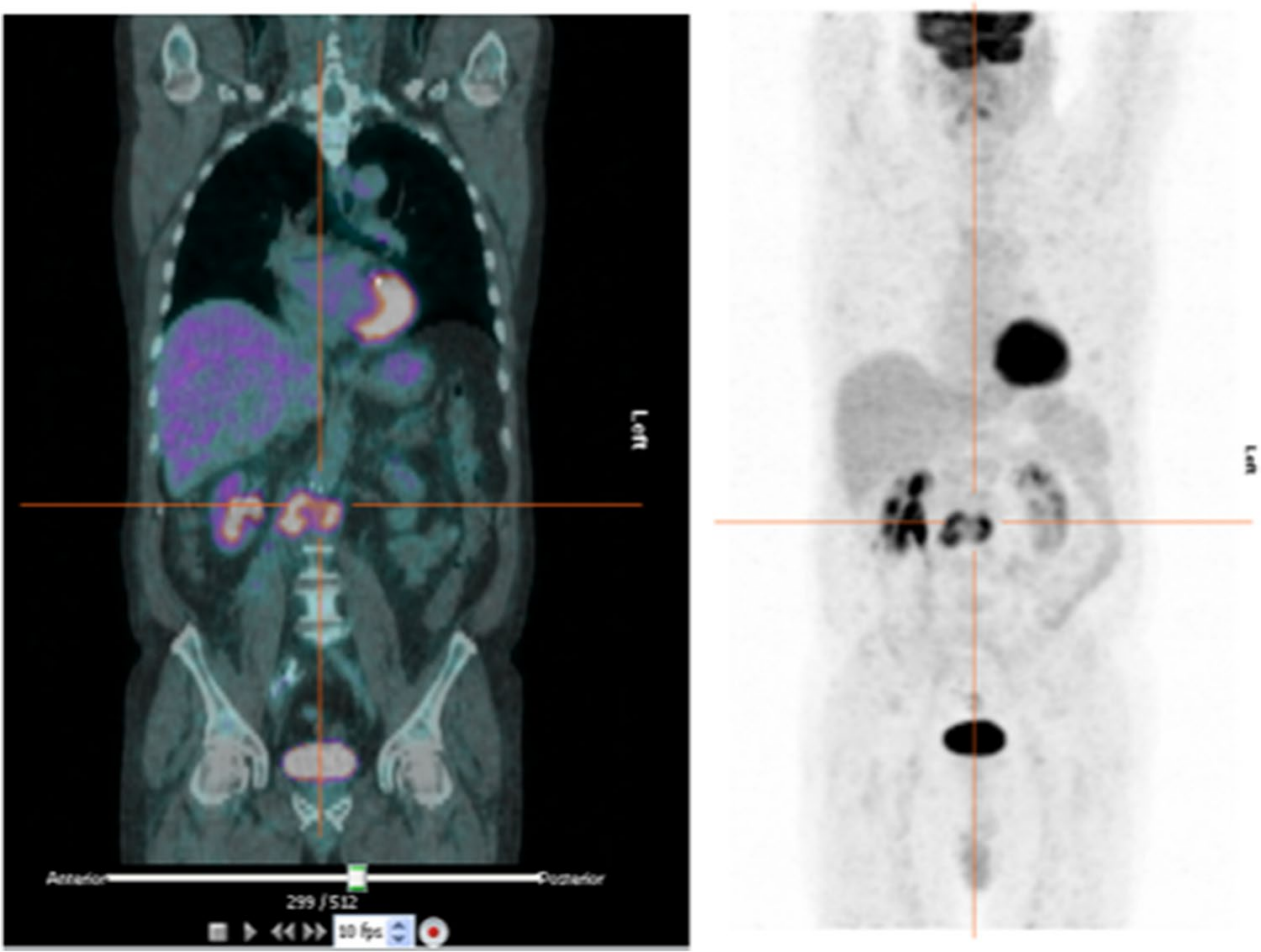
Coronal images of [^18^F]FDG PET/CT (left panel) and MIP (right panel) show focal and intense [^18^F]FDG uptake on the abdominal graft, that is consistent with an infection

Focal uptake was used as criterion of positivity in several studies [28, 31, 33, 38, 49, 53–55, 57, 59, 61–63, 67]. Despite different accuracies have been reported, focal uptake with high [^18^F]FDG intensity, is highly predictive of infection (Fig. 2), whereas moderate, homogeneous, and linear uptake is not. This also emerges from the study published by Keidar et al. in 2014. Aiming to better understand the normal biodistribution of [^18^F]FDG in non-infected grafts, they retrospectively evaluated 107 grafts of different materials and examined the pattern of uptake according to time elapsed from surgery. Diffuse (both homogeneous and non-homogeneous) uptake was detected in 92% of non-infected grafts, as a result of persistent foreign body reaction, and none of them demonstrated focal uptake, thus concluding that diffuse uptake should be interpreted as non-infected [30].

However, some authors underlined that the pattern of [^18^F]FDG uptake in non-infected grafts may be similar to that of infected grafts [38, 57, 66]. Therefore, the evaluation of additional signs of infection (gas bubbles, peri-graft fluid collections, graft dislocation, irregular graft boundaries, fistulae, abscesses) on CT scan is crucial to improve the accuracy of [^18^F]FDG PET, especially in the presence of non-homogeneous uptake [28].

In a recent meta-analysis examining three different interpretation criteria, both focal uptake and SUVmax provided higher sensitivity (93% and 98%, respectively) and specificity (78% and 80%, respectively) compared to visual scoring systems (89% of sensitivity and 61% of specificity) and T/B ratios (57% of sensitivity and 76% of specificity) [48]. However, focal uptake is a qualitative/subjective variable parameter that cannot be easily standardized and SUVmax measurements are not reproducible in all centres. Moreover, as previously mentioned, precise cut-off values for SUVmax or T/B ratios able to discriminate between low-grade or high-grade infections and sterile inflammation, have not been defined. Therefore, its role in the diagnostic setting is still unclear but it could be a useful tool in therapy monitoring [40, 55].

Concluding, among the several qualitative and semi-quantitative interpretation criteria for [^18^F]FDG PET/CT, focal uptake seems to be a reliable tool to diagnose the infection as demonstrated by the high positive and negative predictive values reported. The combination of two or more parameters (grading score, focal pattern, SUVmax, T/B ratios) and radiological signs at co-registered CT should be better explored, especially in more complex cases.

**Table:** 

Grade B	Recommendation 7
	Focal [^18^F]FDG uptake is a reliable tool for differentiating an infection from a sterile post-surgical inflammation or foreign body reaction

8. ***In case of clinical suspicion of VGEI in the early post-surgical phase, CTA is an accurate diagnostic examination.***

**Level of evidence: 5**

Even though the usefulness of CTA examinations in diagnosing VGEI is well known, the accuracy in the immediate post-operative setting (within 7 days) is still unclear. In particular, it isn’t well defined at which time point after surgery the presence of gas, fluid, or peri-aortic fat stranding can be considered suspicious or consistent with VGEI.

For this PICO, no papers published from 2000 to 2021 were retrieved. However, older studies suggested that the presence of peri-graft air bubbles is not inevitably indicative of VGEI. Therefore, in the absence of other signs of infection, the patient must be strictly followed-up with serial CT scans. Any increase in gas volume or fluid collections and the presence of peri-graft air after 4–7 weeks must be considered abnormal [68, 69].

**Fig. 3:**
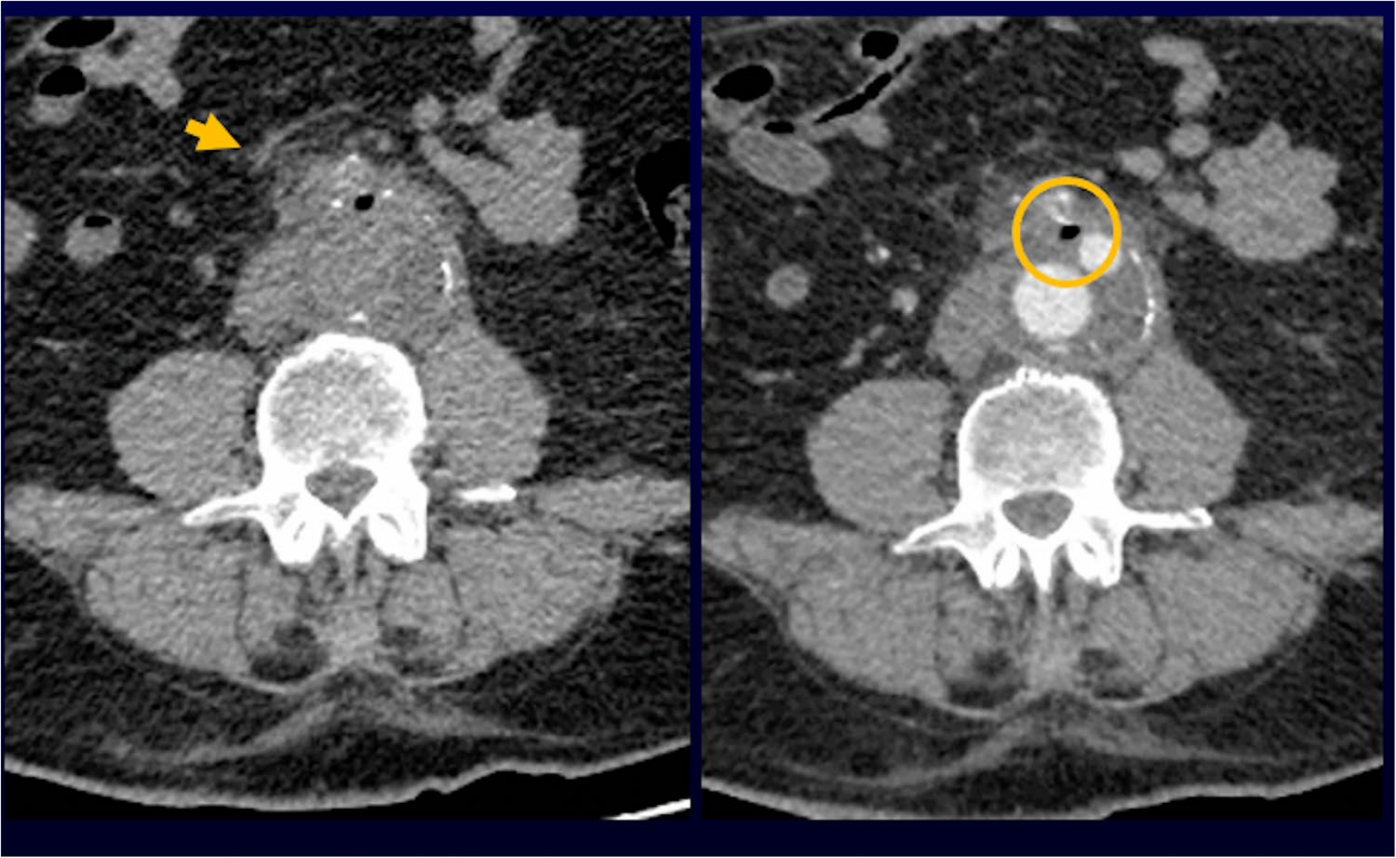
63-yo man who underwent open surgical repair of abdominal aortic aneurysm. CT images (a: unenhanced image and b: arterial-phase image) obtained 4 months after surgical treatment show aortic graft patency with peri-graft soft tissue stranding with peri-graft gas

Boccalini et al. showed that peri-graft fluid collections might be a signal of upcoming complications and require strict follow-up, whereas isolated peri-graft fat stranding up to 17 mm could be considered as a normal post-operative finding [70]. Nevertheless, other authors suggest that the persistence of peri-graft soft tissue stranding after 3 months from surgery, should arise the suspicion for VGEI [71, 72] (Fig. 3).

In conclusion, there is no recent evidence that CTA is the most accurate diagnostic tool in the immediate post-operative phase, and studies from the 80’s and 90’s provide no sufficient information to support this statement, mostly due to small study populations. However, peri-graft air within 7 days or peri-aortic fat stranding and fluid within 3 months can be considered physiological post-surgical findings, only requiring serial CTA follow-up. Peri-graft fluid/gas increasing over time (or persisting after 3 months) or an expansion of the aneurysm sac in the follow-up studies, increase the probability of VGEI. In equivocal cases, or to confirm a positive finding especially in the early post-surgical phase, the appeal to more specific imaging modalities is mandatory. This is particularly important to exclude false positive results at CTA due to sterile inflammation, especially when clinical presentation or laboratory findings are vague and the probability of an infection is low.

**Table:** 

Grade D	Recommendation 8
	CTA has low accuracy in diagnosing VGEI in the early post-surgical phase. In case of doubtful CTA or to confirm positive findings, more specific imaging modalities are needed

9. ***WBC scintigraphy is an accurate technique to diagnose VGEI both in early and late post-surgical phases.***

**Level of evidence: 3**

WBC scintigraphy is the gold standard nuclear medicine method for differentiating between infection and inflammation, as emerges from several published consensus on different infectious diseases. Time interval between surgery and WBC imaging does not influence the results, making WBC imaging already applicable early after surgery [21, 73, 74]. Convincing evidence that WBC scintigraphy is the most accurate examination in the first 4 months after surgery is lacking since comparative studies are scarce. In a recent systematic review and meta-analysis, it was not possible to extract the time interval between implantation of the graft and imaging [7].

One study from 2006 evaluated WBC scintigraphy in the early perioperative period (3 scans, 1 week before, 1 week after, and 1 month after surgery) in 23 patients with an endovascular graft implantation. No false positive WBC scans were found in the first month after surgery. One major limitation of this study is, however, that presence of infection at the end of the follow-up (14 months) was demonstrated in only one patient, so the usefulness of WBC scintigraphy in the early post-operative phase could not be evaluated [42].

Just recently, a retrospective study was published evaluating WBC scintigraphy and [^18^F]FDG PET/CT in 39 patients with 96 grafts. The diagnostic accuracy of WBC scan was significantly higher than [^18^F]FDG PET/CT (90.6% vs 71.9%). The time from surgery was < 4 months in 11 cases, and > 4 months in the remaining 85 cases. Most false positive scans were found with [^18^F]FDG PET/CT, one in a graft that was placed two and a half months before the scan; no false positive scans were found in early post-operative phase with WBC imaging, [44].

So, in conclusion, WBC scintigraphy can be performed even in the early post-operative phase, given the low number of false positive results reported and the high positive and negative predictive value. Nevertheless, it is not possible to conclude that WBC scintigraphy is the most accurate diagnostic examination within the first 4 months after surgery, since comparative studies with other imaging techniques are lacking.

**Table:** 

Grade B	Recommendation 9
	WBC scintigraphy should be used for confirming VGEI, given its high positive predictive value even in early post-surgical phase

10. ***[***^***18***^***F]FDG PET/CT is more accurate to diagnose VGEI in late post-surgical phase than in early post-surgical phase.***

**Level of evidence: 2**

[^18^F]FDG PET/CT is today extensively used for imaging VGEI, in particular in case of equivocal findings on CT [34, 50]. Sensitivity is high, but specificity is low [44]. The reason for false positive findings on PET/CT is the non-specific uptake of [^18^F]FDG in non-infected, inflammatory tissue that is commonly observed in early post-surgical phases. A recent implanted vascular graft is a well-known cause of reactive [^18^F]FDG uptake around the graft. In particular, within the first 6–8 weeks, the risk of a false positive finding is very high [28–30]. A prolonged time interval between surgery and [^18^F]FDG PET/CT imaging may reduce these findings, but increased metabolic activity may persist for many years, depending on the graft material [30].

For this PICO, a literature search was conducted and 74 papers were retrieved, of which 18 were included for thorough evaluation [30, 32, 33, 38, 44, 52–59, 62, 64, 75–77]. The studies mentioned the number of central and/or peripheral grafts. All included papers concluded that [^18^F]FDG PET/CT in late post-surgical phase is a sensitive imaging method, with some lower specificity, as also confirmed in a recent meta-analysis [50]. At the moment, [^18^F]FDG PET/CT can be performed in both early and late infections, but a particular caution should be observed within the first 4 months from surgery. The definition of standardized interpretation criteria is mandatory for accurately differentiating sterile inflammation from infection.

**Table:** 

Grade B	Recommendation 10
	[^18^F]FDG PET/CT can be used in late post-surgical phase, when the normal sterile inflammation decreases

**Fig. 4:**
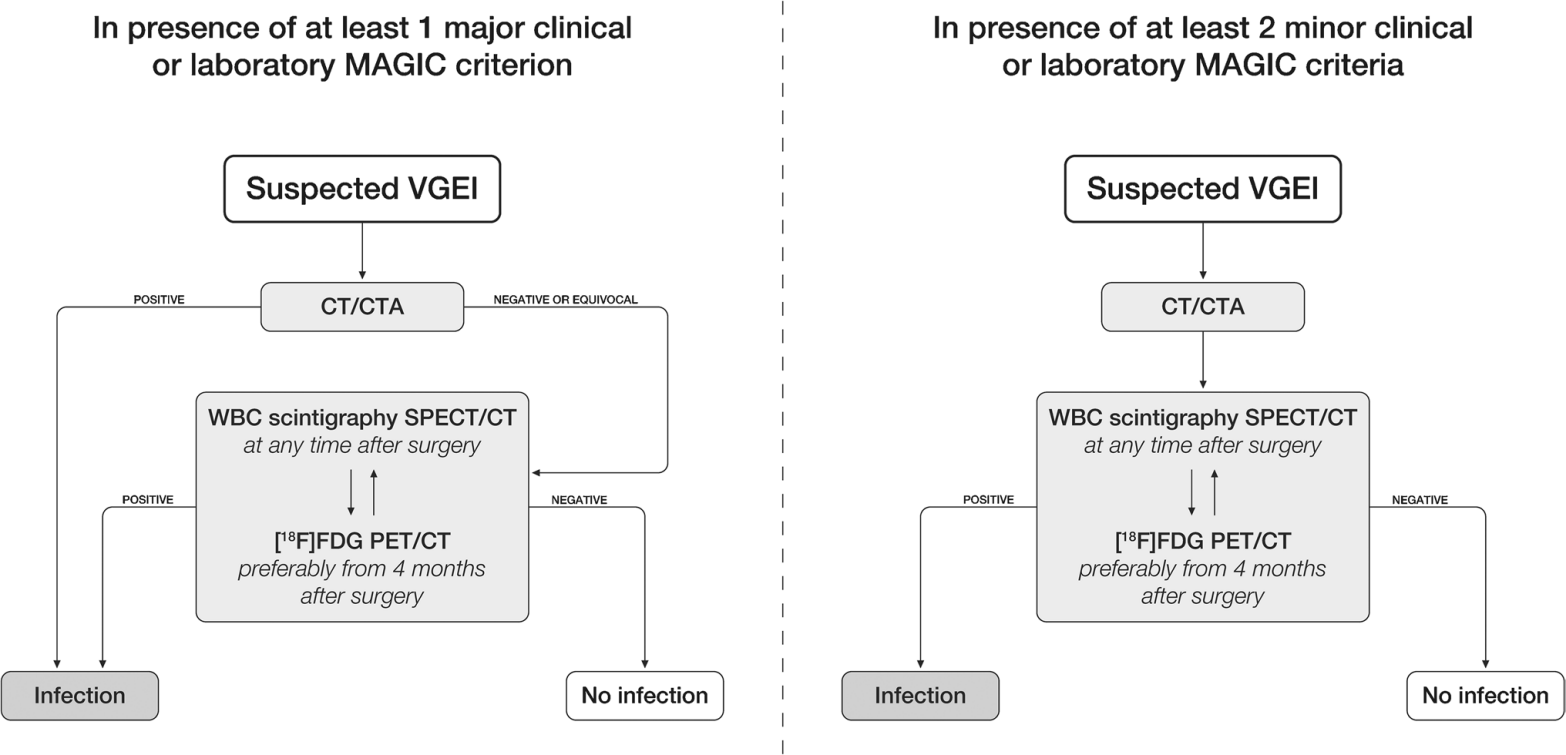
Suggested diagnostic flow charts in suspected VGEI, according to the probability of having an infection prior to imaging. CTA is always the first-line imaging modality. In the presence of at least one major clinical or laboratory MAGIC criterion (higher pre-test probability), a positive CTA is sufficient for the diagnosis of VGEI but, in case of negative or doubtful results with persisting clinical suspicion, NM techniques are strongly recommended. Radiolabelled WBC can be performed at any time after surgery and it should be preferred in the first 4 months after surgery, being more accurate than [^18^F]FDG PET/CT. In late phase after surgery, both WBC scintigraphy and [^18^F]FDG PET/CT can be performed. A negative [^18^F]FDG PET/CT can rule out the infection, but a positive [^18^F]FDG PET/CT result should always be interpreted with caution, and possibly be confirmed by radiolabelled WBC scintigraphy. In the presence of at least two minor clinical or laboratory MAGIC criteria (lower pre-test probability), CTA findings should always be confirmed or rejected with NM modalities (WBC scintigraphy or [^18^F]FDG PET/CT depending on the time from surgery), unless CTA findings are typical of infection (e.g. graft-enteric fistula). In both routes, in case of discordant findings between CTA and NM examination (WBC scintigraphy or [^18^F]FDG PET/CT), the patient should perform an additional NM modality ([^18^F]FDG PET/CT or WBC scintigraphy).

Based on the above-mentioned statements and evidence from the published literature, we developed a diagnostic flow chart for imaging VGEI (Fig. 4). This represents a first attempt for standardization of diagnostic approaches in different centres and will be updated when larger prospective studies, directly comparing different imaging modalities, will be published.

## Practical answers to clinical questions

Based on these statements and PICOs (summarized in Table 5), the questions formulated by the vascular surgeons—as mentioned earlier—can now be answered based on scientific evidence.

- How to differentiate a physiological from a pathological [^18^F]FDG uptake, especially in the early post-operative period?

**Table 5:** Summary table of statements and recommendations

N	Statement	Recommendation	References
1	CTA represents a valuable tool in diagnosing VGEI, despite a wide range of sensitivity and specificity	Due to low sensitivity and moderate accuracy of CTA for low-grade infective processes, NM modalities are recommended in negative or equivocal CTA and persisting suspicion of VGEI	[1, 7, 22, 31, 37–39]
	Level of evidence 2	Grade B	
2	MRI has low accuracy for VGEI	MRI has low accuracy for diagnosing VGEI, especially for chronic or late infections, and is not recommended as first imaging choice	[9, 10, 17, 40, 41]
	Level of evidence 4	Grade C	
3	WBC scintigraphy has high diagnostic accuracy in differentiating VGEI from post-surgical inflammation	WBC scan with SPECT/CT may be used to accurately differentiate an infection from a sterile inflammation	[7, 19–22, 42–47]
	Level of evidence 2	Grade B	
4	Antibiotic therapy has no influence on diagnostic accuracy of WBC scintigraphy in detecting VGEI	No definitive conclusion can be reached in the literature to withdraw or continue antibiotic therapy prior to WBC scintigraphy. The single clinical case should be discussed multidisciplinary	[22, 43, 44]
	Level of evidence 4	Grade C	
5	[^18^F]FDG PET/CT has high sensitivity for diagnosing VGEI	[^18^F]FDG PET/CT has high sensitivity in diagnosing VGEI, regardless of the interpretation criteria used. Therefore, it can be used to rule out the infection	[7, 48–52]
	Level of evidence 2	Grade B	
6	Antibiotic therapy may influence the diagnostic accuracy of [^18^F]FDG PET/CT in detecting VGEI	More robust studies are needed to confirm this effect. The choice to stop or continue antibiotic treatment depends on the single clinical case, preferably discussed within a multidisciplinary team	[7, 48–50, 53–56]
	Level of evidence 3	Grade C	
7	Focal [^18^F]FDG uptake is a reliable diagnostic tool to diagnose an infection	Focal [^18^F]FDG uptake is a reliable tool for differentiating an infection from a sterile post-surgical inflammation or foreign body reaction	[28–33, 37, 38, 44, 49, 52–67]
	Level of evidence 2	Grade B	
8	In case of clinical suspicion of VGEI in the early post-surgical phase, CTA is an accurate diagnostic examination	CTA has low accuracy in diagnosing VGEI in the early post-surgical phase. In case of doubtful CTA or to confirm positive findings, more specific imaging modalities are needed	[68–72]
	Level of evidence 5	Grade D	
9	WBC scintigraphy is an accurate technique to diagnose VGEI both in early and late post-surgical phases	WBC scintigraphy should be used for confirming VGEI, given its high positive predictive value even in early post-surgical phase	[7, 21, 42, 44, 73, 74]
	Level of evidence 3	Grade B	
10	[^18^F]FDG PET/CT is more accurate to diagnose VGEI in late post-surgical phase than in early post-surgical phase	[^18^F]FDG PET/CT can be used in late post-surgical phase, when the normal sterile inflammation decreases	[28–30, 32–34, 38, 44, 50, 52–59, 62, 64, 75–77]
	Level of evidence 2	Grade B	

Since [^18^F]FDG PET imaging is based on the uptake of radioactive glucose in cells/tissue with enhanced metabolism, a physiological non-specific uptake is generally observed in the early perioperative period due to the presence of inflammation. At this time point, given the low accuracy of CTA, WBC scintigraphy should be preferred to [^18^F]FDG PET/CT.

- Is it possible to assess if the whole graft or only a part of the graft is infected?

Even if imaging may differentiate between peri-graft collection and involvement of the graft material, it would be interesting for the clinician to know if the whole graft or only a part of the graft is infected (for example a whole aorto-bifemoral bypass or only the femoral part). The surgical procedure would be different and less invasive if only the infected part could be removed. To this aim, the appeal to hybrid imaging allows to define the exact location and extent of the uptake in both WBC scintigraphy and [^18^F]FDG PET/CT.

- Is it possible to differentiate severe from low-grade infection?

Ideally, one might imagine that the degree of uptake would correlate with the severity of infection, thus suggesting a more aggressive therapeutic approach (e.g. surgical explantation of the whole graft, debridement of the infected area, and in situ reconstruction with a non-prosthetic material) in patients showing higher uptake at [^18^F]FDG PET/CT. Minor degree of uptake could be observed both in low-grade infection, that could be treated by antibiotic therapy alone, or by in situ reconstruction with a antimicrobial-soaked prosthetic material, and sterile inflammation. In practice, there are no scientific data supporting this hypothesis. Moreover, very few data exist on the relationship between [^18^F]FDG PET/CT avidity and prosthetic material.

- Is it possible to differentiate between microbial infection and non-microbial peri-graft reaction?

In some situations, no microbes can be detected, even following surgery. This situation is called “peri-graft reaction”, and the affected graft is replaced with another prosthetic material. A combination of [^18^F]FDG PET/CT and WBC scintigraphy may be extremely useful to differentiate between sterile reaction and infected graft, with particular regard to those with severe peri-graft reactions that may require surgical replacement (e.g. when WBC scan is negative and a PET/CT scan is strongly positive with and [^18^F]FDG uniformly distributed around the graft).

- Is it possible to differentiate between an infection of the graft and the native aortic wall?

Differentiation between mycotic and inflammatory aortic aneurysms is not possible in all cases, especially if sampling of the aortic wall is not possible. In case of stent-graft implantation in a mycotic aortic aneurysm, the possibility of a VGEI must be considered.

In inflammatory aneurysms, post-operative increased [^18^F]FDG uptake of the aneurysm wall may mimic a VGEI (especially in small aneurysm diameters) and result in permanent antibiotic treatment or serial imaging, which may not be necessary. A distinction between [^18^F]FDG uptake of the stent graft and uptake of the aneurysm wall may be beneficial from a clinical point of view, and it is usually achievable by hybrid imaging. The evaluation of co-registered CT scan is, indeed, crucial for the exact definition of the anatomic structures involved by the inflammatory/infective process (Fig. 4).

**Fig. 5:**
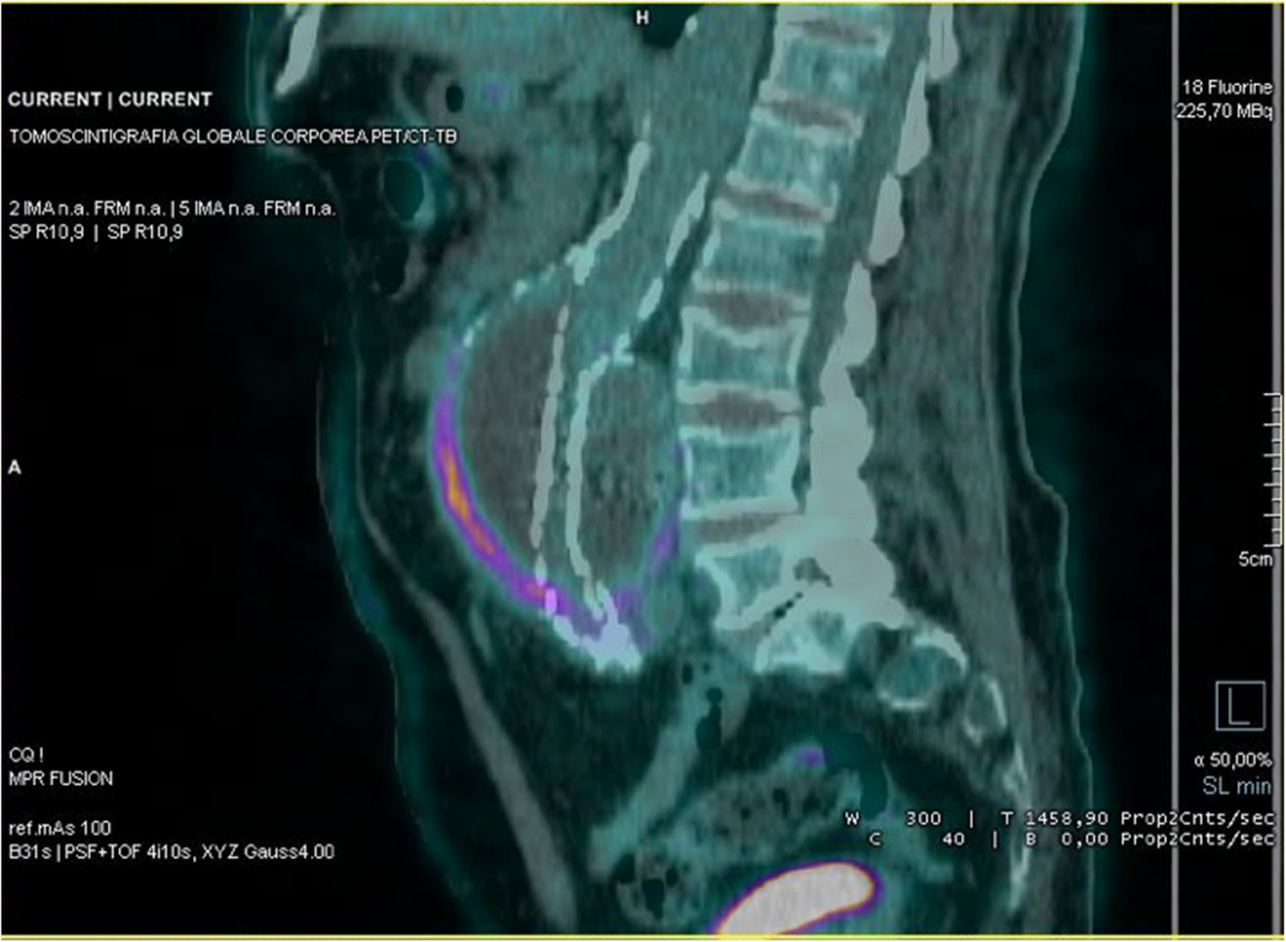
Sagittal view of [^18^F]FDG PET/CT shows mild and homogeneous [^18^F]FDG uptake around the aneurysmatic sac without endograft involvement

## Future research agenda

From the analysis of the current literature, it emerged that several issues are not yet fully covered.

*Clinical issues:*

- Head-to-head comparisons between different imaging modalities are still lacking. Nevertheless, comparing the accuracy of radiolabelled WBC, [^18^F]FDG PET/CT, CTA, and, possibly, MRI in the same patient with histology/microbiology as the gold standard, would be optimal to define the best diagnostic strategy.
- Studied populations are often mixed and comprising patients with both endoprostheses and grafts with different synthetic materials, both intracavitary and extracavitary. It would be desirable to explore the role of imaging in different kinds of vascular grafts, in different synthetic materials, and in different locations (peripheral vs central grafts) to better define which examination is more appropriate in different clinical scenarios. With this effort, larger multicentre prospective studies would be beneficial.
- Imaging is also important in the follow-up of patients, although solid data is needed from comparative longitudinal studies on the evolution of the infection and the sterile post-surgical inflammation. Unknown is why some patients show intense and persisting peri-graft inflammatory reaction, while others do not. Also studies investigating the possible role of antibiotic therapy on NM imaging modalities are lacking.

*Methodological issues:*

- Standardization of acquisition protocols and interpretation criteria of all imaging modalities is warranted to reduce the procedural heterogeneity and to allow optimal comparison between studies. This is particularly important for CTA, MRI, and [^18^F]FDG PET/CT in order to further improve their accuracy in differentiating sterile inflammation from infection.
- Validation of quantitative techniques for imaging with MRI and CTA.
- Validation of new specific protocols and sequences for MRI studies.
- Standardization of visual grading scores, patterns of uptake, and semi-quantitative measurements is crucial to harmonize interpretation criteria of [^18^F]FDG PET/CT between centres.
- To better explore the role of late acquisitions (e.g. after 90–120 min from [^18^F]FDG injection) and dual-time-point PET/CT imaging for improving image quality and T/B ratios.
- To investigate the added value of CTA coregistration in a PET study.
- To investigate the possible role of PET/MRI in imaging VGEI.

*Technical issues:*

- Application of more specific radiopharmaceuticals for infection imaging.
- Evolution of CT Spectral Imaging with better tissue discrimination.
- Application of ECG gated free-breathing phase contrast 3D volume MRA in clinical setting.

Future research should be directed to better explore these topics. It would increase the knowledge in this field and provide more evidence that could be helpful in clinical practice for the management of specific clinical scenarios. Moreover, the availability, in each hospital, of a multidisciplinary team, that jointly analyses cases of suspected VGEI, is crucial to improve patient care (Fig. 5).

## Conclusions and final recommendations

The management of patients with a suspected VGEI highly depends on imaging findings but the diagnosis remains challenging. On the other hand, the removal of an infected graft is a high-risk procedure that needs to be justified by a correct diagnosis. Radiologists and NM specialists should collaborate closely with vascular surgeons, since imaging will help to plan the best management for the patient.

As mentioned before, CTA is the first-line imaging modality in the work-up of patients with suspected VGEI, but the combination with NM techniques is strongly recommended. In particular, in the presence of at least one major clinical or laboratory MAGIC criterion (higher pre-test probability), a positive CTA is sufficient for the diagnosis, but a negative or doubtful scan should always be followed by a NM modality. In the presence of at least two minor clinical or laboratory MAGIC criteria (lower pre-test probability), also a positive CTA should be confirmed by NM modalities, given the low accuracy in differentiating sterile inflammation from infection.

From a NM point of view, both radiolabelled WBC scintigraphy and [^18^F]FDG PET/CT have some pros and cons. Radiolabelled WBC scan are more accurate than [^18^F]FDG PET/CT in the early phase after surgery, while both WBC scintigraphy and [^18^F]FDG PET/CT can be performed in the late phase after surgery. A negative [^18^F]FDG PET/CT can rule out VGEI, due to its high negative predictive value, but a positive [^18^F]FDG PET/CT result should always be interpreted with caution, because of a possible persisting sterile inflammatory reaction.

The choice of an imaging modality should depend on several aspects: the clinical status of the patients and their compliance, local availability and expertise, time elapsed from surgery, results of previous examinations, and waiting lists. Moreover, it also depends on the purpose of the study: if high specificity is required, for example for diagnosing the infection immediately after surgery, then radiolabelled WBC scintigraphy should be preferred; if high sensitivity is needed, for example for the assessment of residual infection after medical treatment, or for the evaluation of late infections, [^18^F]FDG PET/CT could be the technique of choice.

In any case, the decision should derive from a multidisciplinary discussion with all professionals involved in the care of these patients in order to better understand the specific clinical needs. This approach would allow planning the most appropriate diagnostic and therapeutic management for each individual patient.

## Supplementary Information

Below is the link to the electronic supplementary material.

Supplementary file1 (DOCX 37 KB)

Supplementary file2 (DOC 35 KB)

## Abbreviations

CT: Computed tomography
CTA: CT angiography
EANM: European Association of Nuclear Medicine
ESVS: European Society for Vascular Surgery
[^18^F]FDG: 2-Deoxy-2-[^18^F]fluoro-D-glucose
HMPAO: Hexamethylpropylene-amine oxime
MRA: Magnetic Resonance Angiography
MRI: Magnetic resonance imaging
NM: Nuclear Medicine
OCEBM: Oxford Centre for Evidence-based medicine
PET: Positron emission tomography
PICO: Population/problem–Intervention/Indicator–Comparator–Outcome
SPECT: Single-photon emission computerized tomography
SUV: Standardized uptake value
[99 m]Tc: Technetium
T/B: Target-to-background ratio
VGEI: Vascular graft/endograft infection
WBC: White blood cells

## Declarations

### Liability statement

This Guideline summarizes the views of the EANM Inflammation & Infection Committee. It reflects views for which the EANM cannot be held responsible. This Guideline should be taken into context of good practice of nuclear medicine and do not substitute for national and international legal or regulatory provisions.
