# Supplementary material for: Evidence-based guideline of the European Association of Nuclear Medicine (EANM) on imaging infection in vascular grafts
Source: Eur J Nucl Med Mol Imaging. 2022 Apr 4;49(10):3430–51. doi: 10.1007/s00259-022-05769-x (PMC9308572; doi:10.1007/s00259-022-05769-x)
Supplement: Supplementary file 1 — Supplementary file1 (DOCX 37 KB) [file 259_2022_5769_MOESM1_ESM.docx]

# **APPENDIX 1**

**1. CTA represents a valuable tool in diagnosing VGEI, despite a wide range of sensitivity and specificity**

Level of evidence: 2

P: patients with vascular graft infection and complications

I: CT angiography

C: other diagnostic modalities

O:  diagnostic accuracy of CT angiography

Search terms: ((CT) OR (computed tomography) OR (CT angiography) OR (CTA)) AND (vascular) AND ((infect*) OR (pathogenicity)) AND ((graft) OR (bypass) OR (by-pass) OR (prosth*) OR (endoprosth*)) AND ((detect*) OR (diagnos*) OR (accuracy) OR (sensitivity) OR (specificity)) NOT ((review) OR (case report))

All together: 390

Included papers for thorough reading: 100

Included papers after thorough reading: 7

**2. MRI has low accuracy for VGEI.**

Level of evidence: 4

P: patients with vascular graft infection (surgery, bacteriology, indium labelled WBC)
I: MRI
C: no MRI
O: diagnostic accuracy of MRI

Search terms: vasc* AND "graft infection" AND ("magnetic resonance" OR MR) AND ((detect*) OR (diagnos*) OR (accuracy) OR (sensitivity) OR (specificity))

All together: 39

Included papers for thorough reading: 6

Included papers after thorough reading: 5

**3. WBC scintigraphy has high diagnostic accuracy in differentiating VGEI from post-surgical inflammation.**

Level of evidence: 2

P: patients with vascular graft infection

I: white blood cell scan OR leukocyte scan OR WBC OR WBC scintigraphy in patients under antibiotic therapy

C: -

O: diagnostic accuracy of white blood cell scan OR leukocyte scan OR WBC OR WBC scintigraphy

Search terms: ((patients with vascular graft infection AND (2000:2021[pdat])) AND (white blood cell scan OR leukocyte scan OR WBC OR WBC scintigraphy AND (2000:2021[pdat]))) AND (diagnostic accuracy of white blood cell scan OR leukocyte scan OR WBC OR WBC scintigraphy AND (2000:2021[pdat]))

All together: 80

Included papers for thorough reading: 12

Included papers after thorough reading: 8

**4. Antibiotic therapy has no influence on diagnostic accuracy of WBC scintigraphy in detecting VGEI.**

Level of evidence: 4

P: patients with vascular graft infection

I: white blood cell scan OR leukocyte scan OR WBC OR WBC scintigraphy in patients under antibiotic therapy

C: white blood cell scan OR leukocyte scan OR WBC OR WBC scintigraphy in patients without antibiotic therapy

O: diagnostic accuracy of white blood cell scan OR leukocyte scan OR WBC OR WBC scintigraphy

Search terms: ((((vascular graft) OR (bypass) infection) AND (hmpao or oxine or 111In- or 99mTc or technetium-99m or indium-111 or label* white blood cells or leucocytes or WBC or SPECT/CT)) AND ((antibiotic) OR (antimicrobial) OR (antibacterial) OR (antiseptic) OR (germicidal) OR (microbicidal))) AND ((detect*) OR (diagnos*) OR (accuracy) OR (sensitivity) OR (specificity))

All together: 73

Included papers for thorough reading: 18

Included papers after thorough reading: 3

**5. [^18^F]FDG PET/CT has high sensitivity for diagnosing VGEI.**

Level of evidence: 2

P: patients with suspicious vascular graft infection

I: FDG-PET/CT

C: none or other imaging methods

O: sensitivity of FDG-PET/CT

Search terms: ((PET) OR (positron) OR (FDG)) AND ((graft) OR (bypass)) AND (sensitivity)

All together: 713 (from 2000 to 2021)

Included papers for thorough reading: 46

Included papers after thorough reading: 33

**6. Antibiotic therapy may influence the diagnostic accuracy of [^18^F]FDG PET/CT in detecting VGEI.**

Level of evidence: 3

P: patients with vascular graft infection

I: FDG PET/CT in patients under antibiotic therapy

C: FDG PET/CT in patients without antibiotic therapy

O: diagnostic accuracy of FDG PET/CT

Search terms: ((PET) OR (positron) OR (FDG)) AND ((antibiotic) OR (antimicrobial) OR (antibacterial) OR (antiseptic) OR (germicidal) OR (microbicidal)) AND ((graft) OR (bypass)) AND ((detect*) OR (diagnos*) OR (accuracy) OR (sensitivity) OR (specificity))

All together: 281

Included papers for thorough reading: 20

Included papers after thorough reading: 8

**7. Focal [^18^F]FDG uptake is a reliable diagnostic tool to diagnose an infection.**

Level of evidence: 2

P: patients with vascular graft infection

I: pattern of FDG PET/CT OR (positron) OR (PET) OR (FDG)

C:

O: (detect*) OR (diagnos*) OR (accuracy) OR (sensitivity) OR (specificity)

Search terms: ((patients with vascular graft infection AND (2000:2021[pdat])) AND (pattern of FDG PET/CT OR (positron) OR (PET) OR (FDG) AND (2000:2021[pdat]))) AND ((detect*) OR (diagnos*) OR (accuracy) OR (sensitivity) OR (specificity) AND (2000:2021[pdat]))

All together: 109

Included papers for thorough reading: 35

Included papers after thorough reading: 26

**8. In case of clinical suspicion of VGEI in the early post-surgical phase, CTA is an accurate diagnostic examination.**

Level of evidence: 5

P: patients with vascular graft infection within 1 month from surgery

I: CT angiography

C: other diagnostic modalities

O: diagnostic accuracy of CT angiography

Search terms: ((CT) OR (computed tomography) OR (CT angiography) OR (CTA)) AND (vascular) AND ((infect*) OR (pathogenicity)) AND ((graft) OR (bypass) OR (by-pass) OR (prosth*) OR (endoprosth*)) AND ((detect*) OR (diagnos*) OR (accuracy) OR (sensitivity) OR (specificity)) NOT ((review) OR (case report))

All together: 390

Included papers for thorough reading: 100

Included papers after thorough reading: 0

**9. WBC scintigraphy is an accurate technique to diagnose VGEI both in early and late post-surgical phases.**

Level of evidence: 3

P: patients with vascular graft infection

I: < 4 months after surgery

C: > 4 months after surgery

O: diagnostic accuracy

Search terms: ((((vascular) AND (infect*) AND ((graft) OR (bypass) OR (prosth*) OR (endoprosthesis))) AND ((hmpao OR oxine OR 111In- OR 99mTc OR technetium-99m OR indium-111 OR label* white blood cells OR leucocytes OR WBC OR SPECT/CT)))) AND (((PET) OR (positron) OR (FDG) OR (radiology)))) AND (((detect*) OR (diagnos*) OR (accuracy) OR (sensitivity) OR (specificity)) NOT ((review) OR (case report)))

All together: 41

Included papers for thorough reading: 17

Included papers after thorough reading: 8

**10. [^18^F]FDG PET/CT is more accurate to diagnose VGEI in late post-surgical phase than in early post-surgical phase.**

Level of evidence: 2

P: patients with vascular graft infection

I: > 4 months after surgery

C: < 4 months after surgery

O: diagnostic accuracy of FDG PET/CT

Search terms: ((PET) OR (positron) OR (FDG)) AND (vascular) AND (infect*) AND ((graft) OR (bypass) OR (prosth*) OR (endoprosthesis)) AND ((detect*) OR (diagnos*) OR (accuracy) OR (sensitivity) OR (specificity)) NOT ((review) OR (case report)).

All together: 74

Included papers for thorough reading: 33

Included papers after thorough reading: 18
