# Supplementary material for: Evidence-based guideline of the European Association of Nuclear Medicine (EANM) on imaging infection in vascular grafts
Source: Eur J Nucl Med Mol Imaging. 2022 Apr 4;49(10):3430–51. doi: 10.1007/s00259-022-05769-x (PMC9308572; doi:10.1007/s00259-022-05769-x)
Supplement: Supplementary file 2 — Supplementary file2 (DOC 35 KB) [file 259_2022_5769_MOESM2_ESM.doc]

| **Authors and Year** | **Focus of the analysis** | **Articles included** | **Pooled sensitivity (95%CI)** | **Pooled specificity (95%CI)** | **Pooled positive likelihood ratio (95%CI)** | **Pooled negative likelihood ratio (95%CI)** | **Pooled diagnostic odds ratio (95%CI)** |
| --- | --- | --- | --- | --- | --- | --- | --- |
| Reinders Folmer *et al.*  2020 | uptake intensity | 6 | 90% (79-96) | 59% (38-78) | NR | NR | 10.7 (3.4-33.6) |
|  | uptake pattern | 7 | 94% (89-97) | 81% (71-88) | NR | NR | 52.4 (19.4-141.6) |
|  | semi-quantitative analysis* | 6 | 95% (76-99) | 77% (63-87) | NR | NR | 30.9 (7.3-130.8) |
| Kim *et al. 2019* | PET and PET/CT | 10 | 96% (89-98) | 74% (67-81) | 3.7 (2.9-4.9) | 0.06 (0.02-0.15) | 63 (23-173) |
| Rojoa *et al. 2019* | uptake intensity | 6 | 89% (73-96) | 61% (48-74) | 2.32 (1.27-4.22) | 0.17 (0.06-0.53) | 13.2 (3.1-56.6) |
|  | uptake pattern | 7 | 93% (83-97) | 78% (53-92) | 4.3 (1.7-10.9) | 0.89 (0.03-0.25) | 48.7 (9.6-246) |
|  | semi-quantitative analysis* | 6 | 98% (42-99) | 80% (70-88) | 4.98 (3.12-7.94) | 0.03 (0-1.54) | 176.7 (3-10479) |
| Reinders Folmer *et al. 2018* | PET only | 4 | 94% (88-98) | 70% (59-79) | NR | NR | 28.4 (7.8-102.7) |
|  | PET/CT | 5 | 95% (87-99) | 80% (69-89) | NR | NR | 38 (8.5-170.4) |

**Table 1 Supplementary material:** Published meta-analyses about the diagnostic performance of [18F]FDG PET/CT in VGEI.

Legend: 95%CI = 95% confidence interval; NR = not reported; * = semiquantitative analysis by using the SUVmax.
